# Long-chain fatty acid β-oxidation regulates embryonic development by H3K18 acetylation in mice

**DOI:** 10.3389/fcell.2025.1683028

**Published:** 2025-12-03

**Authors:** Kefan Zheng, Hongdi Cui, Ziheng Tang, Entong Song, Qingran Kong, Jiaming Zhang, Hao Li, Qi Zhao

**Affiliations:** 1 Zhejiang Provincial Key Laboratory of Medical Genetics, Key Laboratory of Laboratory Medicine, Ministry of Education, School of Laboratory Medicine and Life Sciences, Wenzhou Medical University, Wenzhou, Zhejiang, China; 2 Ningbo Medical Center, LiHuiLi Hospital, Ningbo, China; 3 Northeastern University, Boston, MA, United States

**Keywords:** fatty, acid, β-oxidation, H3K18Ac, preimplantation, development, metabolic-epigenetic crosstalk, cell cycle arrest

## Abstract

Fatty acids are not only important as energy sources, but also playing crucial roles in maintaining cellular homeostasis. However, their role in preimplantation development remains unclear. Here, we find that fatty acids gradually accumulate after 4-cell stage in mouse preimplantation development. And, the expressions of fatty acid degradation-related genes are increased along the developmental process. Inhibition of long-chain fatty acid β-oxidation (LCFAO) results in preimplantation developmental arrest, downregulated expressions of S phase-related genes, and loss of H3K18ac modification. By profiling the landscape of H3K18ac, we show that H3K18ac is enriched on the promoter regions of S phase-related genes and correlates with their expression. Together, these findings suggest that LCFAO regulate mouse preimplantation development through H3K18ac, providing additional evidence for metabolic-epigenetic crosstalk in embryonic development.

## 1 Introduction

Preimplantation embryonic development starts from zygote and undergoes a series of complex cell division and differentiation processes (Chazaud and Yamanaka, 2016; Mu et al., 2022). In mammals‌, the zygote develops within the oviduct into 2-cell, 4-cell, and 8-cell embryos, ultimately forming a morula and then a blastocyst, which subsequently completes endometrial implantation in the uterus (Clift and Schuh, 2013; Yao et al., 2019). The cell metabolism with dynamic changes plays a crucial role in controlling preimplantation embryonic development. For example, metabolic intermediates can serve as substrates for epigenetic modification enzymes to regulate epigenetic reprogramming, which is essential for early embryonic development (Mirzadeh Azad et al., 2023). Such metabolic-epigenetic coupling mechanism offers a new perspective for understanding the precise regulation of early embryonic development.

Recent advances in metabolic profiling have provided powerful information for deciphering the interaction between cell metabolism and epigenetics. The Jin Zhang group, by profiling the metabolomes of 2-cell embryos and blastocysts in mice, found that the dynamic balance between the competitive metabolites α-ketoglutarate (α-KG) and 2-hydroxyglutarate (2-HG) drives blastocyst formation by regulating histone demethylation in mice (Zhao et al., 2021). Previously, we have established a metabolome profile across all stages of preimplantation development in mice (Li et al., 2022), and found that NAD^+^/SIRT1-mediated H3K27ac removal enables minor zygotic genome activation (ZGA) exit at the 2-cell stage, which is crucial for mammalian preimplantation embryo development (Li et al., 2022). We have also shown that inhibiting lactate leads to a 2-cell arrest, resulting from major ZGA failure by loss of H3K18lac (Li et al., 2024). These findings suggest that metabolic intermediates could directly engage in epigenetic reprogramming to influence preimplantation embryonic development, which contribute to a more comprehensive understanding of the regulation of metabolic microenvironment.

During the establishment of dynamic metabolic profiles in mouse preimplantation embryos, we observed a progressive accumulation of fatty acids. Fatty acids are not only important as energy sources, but also participate in fundamental processes including cell membrane ‌construction, lipid-soluble vitamin transport and storage, and cell signaling, playing crucial roles in maintaining cellular homeostasis (de Carvalho and Caramujo, 2018). Recently, Jin Zhang’s group has established the dynamic lipid landscapes during mouse and human preimplantation embryonic development, showing that lipid desaturases are required for *in vitro* blastocyst development and implantation (Zhang et al., 2024). And, recent studies also indicate that reduced lipid droplet quantity in embryos leads to developmental arrest (Tatsumi et al., 2018). However, the effect of fatty acid metabolism-mediated epigenetic reprogramming on early embryonic development is unclear. In this study, we find that fatty acids are significantly accumulated in preimplantation embryos. However, the expressions of fatty acid degradation-related genes are increased along the developmental process. The inhibition of LCFAO results in preimplantation developmental retardation and cell cycle delay, which might be led by the loss of H3K18ac at the promoter regions of S phase-related genes. Our findings reveal a critical role of LCFAO in mammalian preimplantation development.

## 2 Materials and methods

### 2.1 Mice

Animal welfare and the experimental procedures adhered to the ethical provisions on the Care and Use of Experimental Animals of Wenzhou Medical University and were approved and authorized by the Animal Experimental Committee of the University (No. xmsq 2023-0118). Animal experiments were performed with 7-week-old ICR mice. Animals were maintained under a 12 h light/dark cycle and provided with food and water *ad libitum* in individually ventilated units.

### 2.2 Embryo collection

Embryos were obtained from superovulated mice. Each female was intraperitoneally injected with 6 IU of pregnant mare serum gonadotropin (PMSG, ProSpec, Cat HOR-272), followed by 5 IU of human chorionic gonadotropin (hCG, ProSpec, Cat HOR-250) 48 h later, and paired with male. For the *in vitro* embryos, time points of post-hCG injection were difined as: 24 h for the late 1-cell stage, 30 h for the early 2-cell stage, 40 h for the middle 2-cell stage, 54 h for the late 2-cell stage, 60 h for the 4-cell stage, 70 h for the 8-cell stage, 78 h for the morula stage, and 94 h for the blastocyst stage.

### 2.3 Lipophagy live-cell imaging

Cells were washed three times with PBS, followed by LD labelling in PBS with BODIPY 493/503 for 10 min. Labelled embryos were then imaged on Lecia SP5 confocal microscope in ibidi μ-plates. Fiji (InageJ) was used to analyze/quantify the volume of lipid droplets from 3D images.

### 2.4 LD staining

The culture medium was carefully removed, and cells were rinsed once with phosphate-buffered saline (PBS) for 5 min at room temperature. Cells were then incubated with 2.5 μM BODIPY 493/503 for 30 min at room temperature in the dark. After staining, cells were washed three times with PBS, each for 5 min. Cells were subsequently washed three times with PBS to reduce background fluorescence. Fluorescence images were captured using a fluorescence microscope.

### 2.5 BrdU staining

First, 10 mM BrdU (1:1000 dilution) was added to the culture medium of 4-cell stage embryos, followed by incubation at 37 °C until they reached the 8-cell stage. The embryos were then washed three times with PBS (5 min each), were fixed for 40 min, and were permeabilized for 20 min. Next, samples were treated with 2M HCl for 30 min at room temperature to denature DNA and expose BrdU epitopes. After blocking for 1 h, they were incubated overnight at 4 °C with a BrdU primary antibody (1:50 dilution). Following three washes with washing buffer, the embryos were incubated for 1 h with a secondary antibody (Goat anti-Mouse 647, Thermo Fisher, A32728, 1:1000 dilution). Finally, nuclei were counterstained with Hoechst for 10–15 min, and the samples were washed three times with washing buffer before imaging.

### 2.6 Immunofluorescence

After having removed the zona pellucida using acidic operating fluid, mouse embryos were fixed in 4% PFA for 40 min at room temperature, followed by permeabilization in 1% Triton X-100 (93443, 100 mL; Sigma) for 20 min at room temperature. Embryos were blocked in blocking solution consisting of 1% bovine serum albumin (BSA) in phosphate-buffered saline (PBS) for 1 h at room temperature after three washes in washing solution (0.1% Tween-20, 0.01% Triton X-100 in PBS). Embryos were incubated with the indicated antibodies against H3K18ac (Abcam, ab40888), overnight at 4 °C. The next day, the embryos were washed in washing solution and incubated with the secondary antibodies (A10040; Invitrogen) for 1 h at room temperature. After staining with Hoechst, the embryos were washed in washing solution and were imaged using an inverted confocal microscope (TCS SP8; Leica, Wetzlar, Germany); image analysis was performed using LAS X software (Leica).

### 2.7 qPCR

RNA was extracted from embryos at various developmental stages (Zygote, 2-Cell, 4-Cell, 8-Cell, Morula, Blastocyst) using the TRIzol Plus RNA Purification Kit (Thermo Fisher). Gene primers were designed as per the gene sequences of Acaa2, Acadl, Acsl5, Hadh, Hadhb, Echs1and Hadha in PubMed Gene and were synthesized by Sangon Biotech (Shanghai) Co., Ltd. Primers were shown in Table 1. Fatty acid inhibitor treatment in embryos.

**TABLE 1:** Primer sequences of mRNA for qRT-PCR.

Primer sequence, 5′-3′	Forward	Reverse
Gene (mouse)		
Acaa2	CTGCTACGAGGTGTGTTCATC	AGCTCTGCATGACATTGCCC
Acadl	TCTTTTCCTCGGAGCATGACA	GACCTCTCTACTCACTTCTCCAG
Acsl5	TCCTGACGTTTGGAACGGC	CTCCCTCAATCCCCACAGAC
Hadh	TCAAGCATGTGACCGTCATCG	TGGATTTTGCCAGGATGTCTTC
Hadhb	ACTACATCAAAATGGGCTCTCAG	AGCAGAAATGGAATGCGGACC
Echs1	TTGTGAACTTGCCATGATGTGT	TGCTCGGGTGAGTCTCTGAG
Hadha	TGCATTTGCCGCAGCTTTAC	GTTGGCCCAGATTTCGTTCA

### 2.8 Fatty acid inhibitor treatment in embryos

We selected three fatty acid inhibitors–Trimetazidine (Merck, PHR1437), Etomoxir (aladdin, E124862), and 2-Methylene cyclopropane acetic acid (MCE, HY-W710928)- to treat embryos starting from the zygote stage. We quantified the percentage of cells not reaching each developmental time point. The inhibitor treatments were initiated at the two-cell stage by addition to the culture medium.

### 2.9 RNA-seq

We performed library construction using Single Cell Full Length mRNA-Amplification (Vazyme, N712) on six biological replicates of embryos at both the 8-cell and morula stages, comparing control and TMZ-treated groups. Briefly, cells were homogenized in 500 μL RL Buffer, followed by DNase I treatment (10 μL, 15 min at RT). Lysates were mixed with equal volume 70% ethanol and loaded onto RNA Spin Columns. After centrifugation (12,000 rpm, 1 min), the columns were washed sequentially with RW1 Buffer and RW2 Buffer (twice), followed by drying centrifugation (12,000 rpm, 2 min). RNA was eluted in 30–50 μL RNase-free water after 2 min incubation.

### 2.10 RNA-seq data quality control, processing, and analysis

RNA-seq data from embryos were analyzed as follows. Quality control of the Illumina reads was performed with FastQC, followed by adapter trimming using fastp. Cleaned reads were then aligned to the GRCm39 reference genome (Ensembl) using the STAR aligner. The raw gene count matrix was generated with FeatureCounts. Differential expression analysis was conducted directly on the raw counts using the R package DESeq2. For purposes of gene expression visualization and comparison within samples, normalized expression levels were also calculated as FPKM (fragments per kilobase of transcript per million mapped reads). Gene Ontology (GO) and KEGG enrichment analyses for the differentially expressed genes identified by DESeq2 were performed with the online tool KOBAS (http://kobas.cbi.pku.edu.cn/kobas3/), and metabolic pathway analysis was performed using PMEA.

### 2.11 CUT&Tag

CUT&Tag was performed using the Hyperactive Universal CUT&Tag Assay Kit for Illumina (TD903, Vazyme Biotech) in accordance with the manufacturer’s instructions. Briefly, 8-Cell and morula stage embryos were collected and counted for incubation with pre-coated concanavalin A beads. Subsequently, cells were permeabilized by digitonin and incubated with H3K18ac antibodies. Next, the samples were incubated with pA-Tn5 transposase. After transposon activation and tagmentation, DNA was isolated, amplified, and purified to construct the library. The samples were then analyzed on an DNBSEQ-T7 platform.

### 2.12 CUT&Tag data analysis

We first assessed the quality of paired-end H3K18ac CUT&Tag sequencing data using FastQC, followed by adapter trimming and quality filtering using Fastp. Then, we aligned the processed reads to the mouse reference genome (genome assembly:mm10) downloaded from the UCSC database using Bowtie2 (v2.5.4). We converted the merged BAM files from replicate samples into BigWig (.bw) format using the bamCoverage tool within the deepTools suite, with RPKM normalization selected as the standardization parameter. Subsequently, we employed the computeMatrix and plotHeatmap tools from the deepTools suite to visualize the intensity profiles of CUT&Tag peaks. Finally, we performed peak calling using MACS3 (v3.0.3), annotated the identified peaks with R package ChIPseeker, and visualized the results in IGV.

### 2.13 Statistical analysis

Statistical analyses were performed using R software (v4.2.1). Data are presented as the mean ± SEM. Specific statistical tests and significance levels are detailed in the respective figure legends.

## 3 Results

### 3.1 Fatty acids are enriched in preimplantation embryos in mice

**FIGURE 1:**
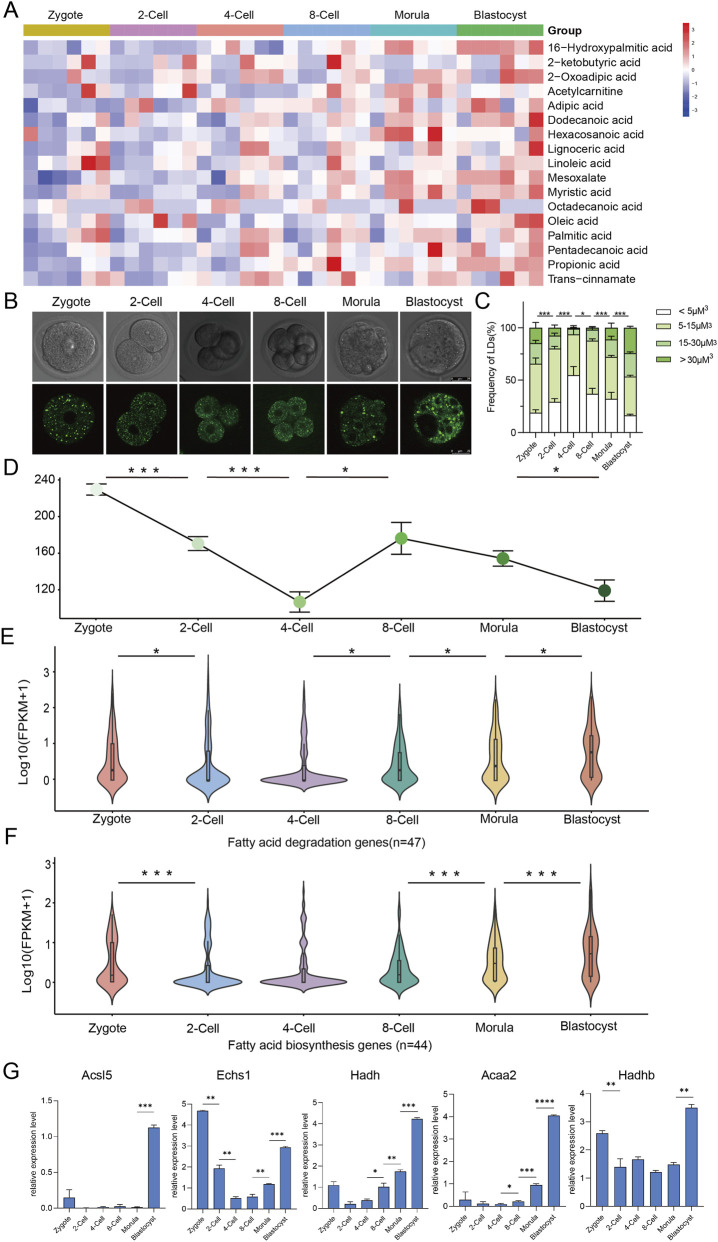
Dynamic metabolic profiles of fatty acid during mouse pre-implantation embryonic development. **(A)** Heatmap showing dynamics of fatty acid metabolites during mouse preimplantation embryonic development. **(B)** Images of BODIPY 493/503-stained lipid droplets (LDs) in in vitro pre-implantation embryos from zygote to blastocyst stages in KSOM medium. Scale bars, 25 μm. **(C)** Quantification of volume of LDs in embryos from **(B)** Error bars, mean ± SEM; Zygote (n = 5 biologically independent replicates), 2-Cell (n = 10), 4-Cell (n = 8), 8-Cell (n = 5), Morula (n = 4), Blastocyst (n = 9); Student’s t-test; *p < 0.05; ***p < 0.001. **(D)** Quantification of the total number of lipid droplets per embryo from **(B)**. Error bars, means ± SEM; two-tail t-test; *p < 0.05; ***p < 0.001. **(E)** Violin plot showing dynamic changes in lipid biosynthesis pathway-associated genes during mouse pre-implantation embryonic development. n = 3 biologically independent replicates; Student’s t-test; *P < 0.05. **(F)** Violin plot showing dynamic changes in lipid degradation pathway-related genes during mouse pre-implantation embryonic development. n = 3 biologically independent replicates; Student’s t-test; ***P < 0.001. **(G)** The expressions of some β-oxidation related genes during mouse pre-implantation embryo development checked by qPCR.Error bars, mean ± SEM; Student’s t-test; *p < 0.05; **p < 0.01, ***p < 0.001.

Our previous metabolomic data showed the significant accumulation of fatty acids beginning at the 8-cell stage (Figure 1A) (Li et al., 2022). To validate that, we measured lipid droplet size, proportion, and number across all stages of preimplantation development in mice, and found that lipid droplet size, the proportion of enlarged droplets, and droplet number all significantly decreased at the 4-cell stage. After the 8-cell stage, although the droplet number showed a decreasing trend, the proportion of larged droplets continued to increase (Figures 1B–D). Lipid metabolism comprises anabolic and catabolic pathways. We further examined transcriptomic data (GSE70605) for genes involved in fatty acid synthesis and degradation, and observed the expressions of genes in both the pathways were significantly upregulated from the 8-cell stage onwards (Figures 1E,F). Given that lipid droplet was accumulated during the process, we indicate the high activation of fatty acid degradation pathway happens in mouse preimplantation embryos. Definitely, we confirmed the high expressions of some key fatty acid β-oxidation-related genes, especially at the blastocyst stage, by qPCR (Figure 1G). These findings suggest that fatty acid β-oxidation may play a crucial role during preimplantation embryonic development in mice.

### 3.2 LCFAO inhibition results in preimplantation developmental arrest in mice

**FIGURE 2:**
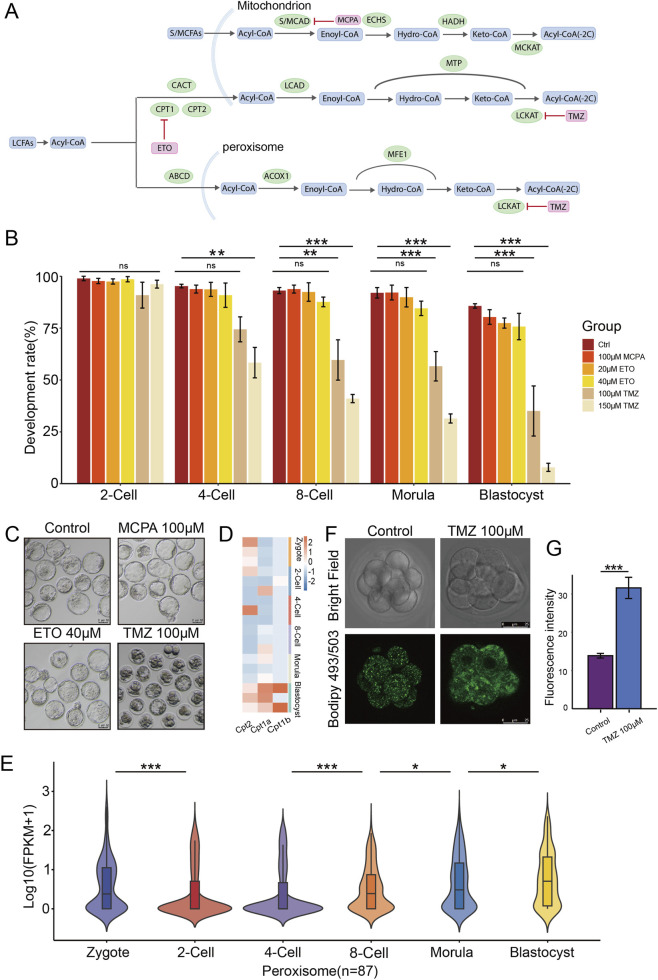
Inhibition of LCFAO results in pre-implantation developmental arrest in mice. **(A)** Schematic diagram of the metabolic pathways of long-chain, and medium-/short-chain fatty acids. **(B)** Bar plots showing the proportion of embryos that successfully developed to each stage under control conditions and treatment with different types and doses of inhibitors. Error bars, means ± SEM; n = 3 biologically independent replicates. ns, not significant; one-way analysis of variance (one-way ANOVA); **p < 0.01; ***p < 0.001. **(C)** Development images of control, MCPA-treated, ETO-treated and TMZ-treated embryos. **(D)** Heatmap showing dynamics of Cpt1a, Cpt1b, and Cpt2 genes expressions during mouse preimplantation embryonic development. **(E)** Violin plot showing the expressions of peroxidase family genes during mouse preimplantation embryonic development. n = 3 biologically independent replicates. **(F)** Images of BODIPY 493/503-stained lipid droplets (LDs) in the control and TMZ-treated 8-cell embryos. Scale bars, 25 μm. **(G)** Quantification of fluorescence intensity from **(F)**. Error bars, means ± SEM; n = 3 biologically independent replicates; Student’s t-test; ***p < 0.001.

Fatty acid β-oxidation primarily occurs in mitochondria and peroxisomes. Fatty acids can be classified into long-chain fatty acids (LCFAs) and medium/short-chain fatty acids (MCFAs/SCFAs) based on the length of carbon chains, and LCFAs require the carnitine acyltransferases CPT1 and CPT2 for the intake into mitochondria (Figure 2A). To investigate the role of fatty acid β-oxidation in preimplantation embryonic development, we treated embryos with different concentrations of Etomoxir (CPT1a inhibitor, ETO), Trimetazidine (long-chain 3-ketoacyl-CoA thiolase inhibitor, TMZ), and 2-Methylene cyclopropane acetic acid (medium/short-chain fatty acid β-oxidation inhibitor, MCPA). We found that MCPA treatment had no significant effect on the early development, which was in line with the point that LCFAs are the most abundant fatty acids in mammalian cells. However, we observed that TMZ treatment, but not ETO, led to massive embryonic arrest, with significantly lower developmental rates at the 8-cell to blastocyst stages compared to the control group (Figures 2B,C). Actually, it has been reported that ETO has no significant effect on preimplantation embryonic development. This may be attributed to the fact that Cpt1a, Cpt1b, and Cpt2 genes are barely expressed before the blastocyst stage (GSE70605) (Figure 2D), and the transcriptomic data (GSE70605) also showed the expressions of peroxidase family genes were particularly high from the 8-cell stage (Figure 2E), indicating that the LCFAO may mainly happen in peroxisomes, but not mitochondria, at the early stages of mouse preimplantation development. In addition, TMZ treatment resulted in a significant enrichment of fatty acids at the 8-cell stage (Figures 2F,G), suggesting that TMZ inhibits the degradation of fatty acids. These results demonstrate that the β-oxidation of LCFAs plays a regulatory role in mouse preimplantation embryonic development.

### 3.3 Inhibition of LCFAO leads to S phase arrest in mouse preimplantation embryos

**FIGURE 3:**
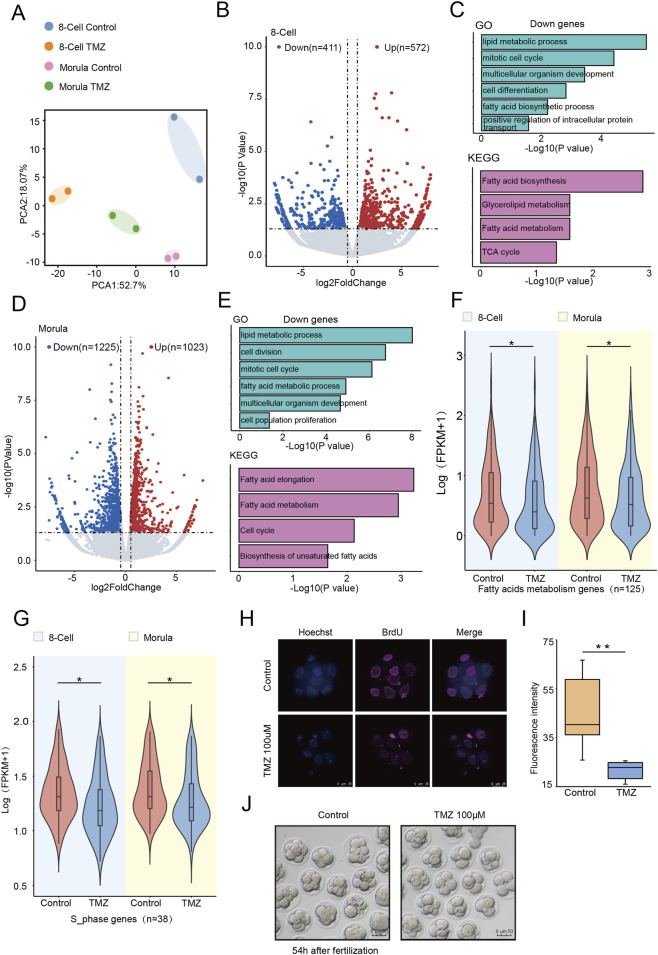
Inhibition of LCFAO leads to cell cycle arrest in mouse preimplantation embryo. **(A)** Principal component analysis (PCA) plots of control and TMZ-treated embryos at the 8-cell and morula stages (n = 2 biologically independent replicates) **(B)** RNA-seq analysis of control and TMZ-treated mouse 8-cell embryos. Volcano plots show gene expression changes. Red and blue dots indicate upregulated (log2FoldChange >0.5) and downregulated (log2FoldChange < −0.5) genes with P < 0.05. **(C)** GO and KEGG analysis of downregulated genes in TMZ-treated 8-cell mouse embryos. **(D)** RNA-seq analysis of control and TMZ-treated mouse morula embryos. Volcano plots show gene expression changes. Red and blue dots indicate upregulated ((log2FoldChange >0.5) and downregulated (log2FoldChange < −0.5) genes with P < 0.05. **(E)** GO and KEGG analysis of downregulated genes in TMZ-treated mouse morula embryos. **(F)** Violin plot shows the downregulation of fatty acid metabolism-related genes in TMZ-treated mouse 8-cell and morula embryos. **(G)** Violin plot shows the downregulation of cell cycle-related genes in TMZ-treated mouse 8-cell and morula embryos. **(H)** BrdU staining showing that TMZ-treated embryos lead to cell division arrest. Scale bars, 25 μm. **(I)** Quantification of fluorescence intensity from **(H)**. Error bars, means ± SEM; n = 6 biologically independent replicates; Student’s t-test; **p < 0.01. **(J)** Developmental images showing TMZ treatment leads to the delayed embryonic development.

To investigate the mechanisms by which LCFAs affect preimplantation development, we performed RNA sequencing (RNA-seq) on 8-cell embryo and morula treated with TMZ. Principal component analysis (PCA) distinguished the TMZ-treated groups from controls (Figure 3A). Differential expression gene analysis revealed that 572 genes were upregulated and 411 genes were downregulated in TMZ-treated 8-cell embryos compared to the control group (Figure 3B). GO analysis indicated that the downregulated genes were enriched in lipid metabolic process, mitotic cell cycle, and fatty acid biosynthetic process, and KEGG analysis were enriched in fatty acid biosynthesis and fatty acid metabolism (Figure 3C). At the morula stage, in comparison to the control group, 1225 genes were upregulated and 1023 genes were downregulated in the treated embryos (Figure 3D), with downregulated genes primarily involved in lipid metabolic process, mitotic cell cycle and fatty acid metabolic process and KEGG analysis were enriched in fatty acid elongation, fatty acid metabolism, and biosynthesis of unsaturated fatty acids (Figure 3E). Furthermore, the expressions of genes related to fatty acid metabolism and cell cycle S phase were downregulated (Figures 3F,G). To validate the impact of TMZ on the S phase, we performed 5-bromodeoxyuridine (BrdU) staining on 8-cell embryos, and found significantly reduced DNA synthesis signals in TMZ-treated embryos (Figure 3H). Furthermore, the developmental delay was also observed in the TMZ-treated embryos at 54 h post-fertilization. These findings suggest that LCFAO participates in regulating cell cycle progression in mouse preimplantation embryos.

### 3.4 Inhibition of LCFAO results in the reduced H3K18ac levels in mouse preimplantation embryos

**FIGURE 4:**
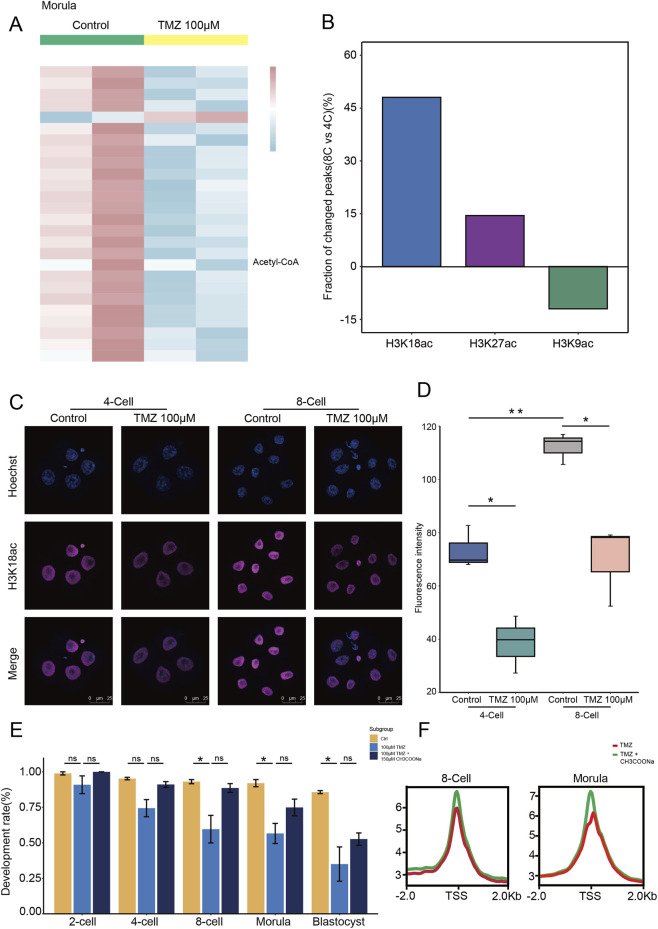
Inhibition of LCFAs β-oxidation leads to a reduction in H3K18ac levels. **(A)** Heatmap showing that compared to the control group, TMZ treatment led to a reduction in fatty acid metabolism-associated intermediates levels. **(B)** Dynamic changes in H3K18ac, H3K27ac, and H3K9ac enrichments from the 4-cell to 8-cell stages. **(C)** Immunofluorescence of H3K18ac on mouse 4-cell and 8-cell embryos with or without TMZ treatment. **(D)** Quantification of fluorescence intensity from **(C)** Error bars, means ± SEM; n = 3 biologically independent replicates; Student’s t-test; *p < 0.05, **p < 0.01. **(E)** Bar plots showing the proportion of embryos that successfully developed to each stage under control conditions, treatment with the TMZ inhibitor, and treatment with the TMZ inhibitor followed by acetate supplementation. Error bars, means ± SEM; n = 3 biologically independent replicates. ns, not significant; one-way analysis of variance (one-way ANOVA); *p < 0.05. **(F)** Metaplot of H3K18ac enrichment at the promoters of coding genes in 8-cell and morula embryos treated with the TMZ inhibitor and with the TMZ inhibitor followed by acetate supplementation.

To understand the cell cycle arrest resulted by the inhibition of LCFAO, we utilized our previously developed preimplantation embryo metabolic analysis computational framework (Preimplantation Embryos Metabolic Analysis, PEMA; available at https://github.com/summus-kong/PEMA) to analyze the transcriptomic data of morulae treated with TMZ. The results indicated that the levels of various fatty acid metabolism-associated intermediates were decreased by TMZ treatment, such as acetyl-CoA levels, which serves as a key substrate for histone acetylation (Figure 4A). Given that the remarkable cell cycle arrest begins at the 8-cell stage, we analyzed the dynamic changes in genome-wide histone modifications (H3K18ac, H3K27ac, and H3K9ac) from the 4-cell to 8-cell stages using publicly available databases (CRA006815). The data revealed a significant increase in H3K18ac levels between the 4-cell and 8-cell stages (Figure 4B). The results were also confirmed by immunofluorescence (Figures 4C,D). Importantly, in the TMZ-treated groups, the acetylation signals of H3K18 were significantly diminished (Figures 4C,D). The addition of sodium acetate can compensate for histone acetylation; therefore, we supplemented sodium acetate together with TMZ. Although the compensatory effect on development was not substantial (Figure 4E), embryos in the acetate-supplemented group showed a slight increase in H3K18ac signal enrichment at both the 8-cell and morula stages compared to the TMZ-treated group (Figure 4F). Acetate supplementation exhibited a moderate compensatory effect. Altogether, these findings indicate that inhibition of LCFAO leads to a reduction in H3K18ac levels.

### 3.5 LCFAO may influence the expression of S phase-related genes through H3K18ac

**FIGURE 5:**
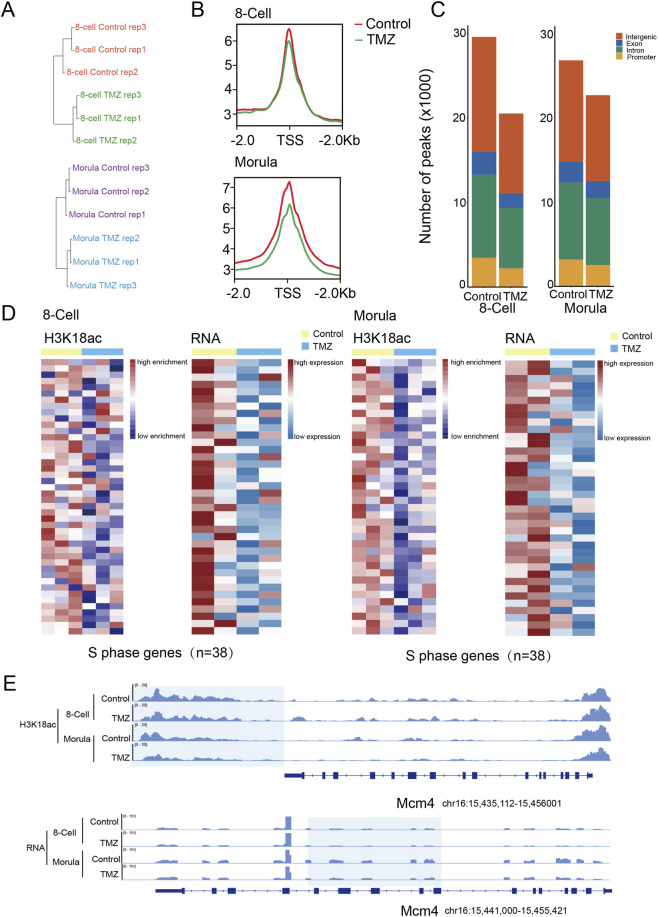
LCFAs β-oxidation regulates the expressions of S phase-related genes by H3K18ac. **(A)** Unsupervised clustering of H3K18ac signals of the embryos as indicated. n = 3 biologically independent replicates **(B)** Metaplot of H3K18ac enrichment at the promoters of coding genes of the embryos as indicated. **(C)** Fraction of the mouse genome covered by H3K18ac reads among the embryos as indicated, and percentages of H3K18ac peaks assigned to the promoter, intron, exon, and intergenic regions. **(D)** Heatmap (left) of H3K18ac signals ranked by their relative changes and expression changes of S phase genes between the 8-cell embryos treated with or without TMZ. Heatmap (right) of H3K18ac signals ranked by their relative changes and expression changes of S phase genes between the morula treated with or without TMZ. **(E)** Genome browser view of H3K18ac (up) and RNA (down) signals at the *Mcm4* locus at the 8-cell and morula embryos.

To understand how H3K18ac mediated by LCFAO regulates mouse preimplantation development, we employed low-input Cleavage Under Targets and Tag mentation (CUT&Tag) technology to establish the genome-wide H3K18ac histone modification profiles in 8-cell and morula embryos. Unsupervised clustering analysis demonstrated high intra-group correlation and clear separation between the control and TMZ-treated groups (Figure 5A). Both the intensity and enrichment of the H3K18ac signals were reduced in TMZ-treated embryos compared to controls at both the 8-cell and morula stages (Figures 5B,C). Notably, TMZ-treated 8-cell and morula embryos exhibited significantly lower H3K18ac peaks at S phase-related genes, which was correlated with their downregulated expressions (Figure 5D). For example, the decreased H3K27ac and RNA signals were observed at the locus of DNA replication licensing factor, Mcm4, in the TMZ-treated group (Figure 5E). The results suggest that the loss of H3K18ac levels at the promoters of S phase-related genes may contribute to the impaired expressions. Collectively, we demonstrate that LCFAO could regulate the cell cycle through H3K18ac and plays a critical role in mouse preimplantation development.

## 4 Discussion

Preimplantation embryonic development is a process involving cell proliferation and epigenetic remodeling (Jukam et al., 2017). This process requires precise regulation of transcription and epigenetic modifications by metabolites to ensure orderly development; otherwise, it may lead to embryonic developmental failure (Zhao et al., 2023). In this study, we observed substantial accumulation of fatty acids in mouse embryos during preimplantation development, and the expressions of fatty acid degradation-related genes are also increased along the developmental process. Inhibition of LCFAO resulted in the developmental retardation at the 8-cell stage. Mechanistically, LCFAO could regulate the expressions of cell cycle S phase-related genes through H3K18ac.

Preimplantation embryos acquire nutrients from oviduct fluid, including pyruvate, lactate, and glucose (Brinster, 1965a; Brinster, 1965b). Beyond these nutrients, embryos also utilize endogenous energy reserves such as fatty acids. Lipids stored in mammalian oocytes serve as potential energy reservoirs before ZGA, with fatty acids accumulating in the cytoplasm via hydrophobic lipid droplets (Sturmey et al., 2009). The quantity and distribution of lipid droplets vary significantly among species. For instance, pig, cow, and sheep oocytes and early embryos contain abundant lipid droplets (Ferguson and Leese, 1999; McEvoy et al., 2000; Leroy et al., 2005; Schelbach et al., 2010; Sudano et al., 2011), whereas mouse and human embryos possess relatively fewer (Matorras et al., 1998; Haggarty et al., 2006; Bradley et al., 2016). In this study, we observed that lipid droplets gradually increase after 4-cell stage in mouse early embryos, becoming particularly pronounced at the morula and blastocyst stages, indicating a progressive elevation in embryo metabolic activity as development proceeds.

Endogenous lipids play a crucial role in mouse embryonic development; reduced lipid droplet numbers correlate with decreased triglyceride levels and embryonic developmental arrest (Tatsumi et al., 2018), and lipid desaturases are required for *in vitro* blastocyst development and implantation (Zhang et al., 2024). However, in the study, we show that fatty acid degradation pathway is upregulated as the preimplantation development proceeds, and inhibiting LCFAO leads to embryonic arrest and significantly reduces blastocyst formation rates, indicating the indispensable role of LCFAO in preimplantation development. LCFAO is essential for energy homeostasis. While the prevailing view suggests that the preimplantation embryos primarily rely on glycolysis for energy, thus an interesting but tangential thought emerges: how LCFAO regulates on preimplantation embryonic development. β-oxidation results in the shortening of fatty acids by two carbons per cycle, generating acetyl-CoA. This study has certain limitations. Although the differential peaks of H3K18ac exhibited the highest proportion at the 4-cell to 8-cell stage,our investigation focused solely on the mechanism linking long-chain fatty acid oxidation to H3k18ac. Our results demonstrate that LCFAO inhibition causes a significant decline in H3K18ac at the locus of S phase-related genes, downregulating their expressions. In summary, this study preliminarily explores the link between fatty acid metabolism and epigenetic reprogramming in mammalian preimplantation embryos, further providing potential metabolic intervention targets for optimizing embryo culture conditions in assisted reproductive technologies.

## Data Availability

Publicly available datasets analyzed in this work are available in GEO and GSA, the GSE ID shown below: GSE70605 (RNA-seq datasets of Mouse early embryos, https://www.ncbi.nlm.nih.gov/search/all/?term=GSE70605), the CRA ID shown below: CRA006815 (CRX413025, CRX413024, CRX413027, CRX413034, CRX413032 and CRX13035) (Cut-tag datasets of Mouse early embryos, https://ngdc.cncb.ac.cn/gsa/search?searchTerm=CRA006815). RNA-seq and CUT&Tag data of mouse embryos generated in this study have been deposited in GSA under accession GSE303695 (https://www.ncbi.nlm.nih.gov/search/all/?term=GSE303695) and GSE304392 (https://www.ncbi.nlm.nih.gov/search/all/?term=GSE304392).

## Abbreviations

LCFAO, long-chain fatty acid β-oxidation; α-KG, α-ketoglutarate; 2-HG, 2-hydroxyglutarate; ZGA, zygotic genome activation; LDs, lipid droplets; LCFAs, long-chain fatty acids; ETO, Etomoxir; TMZ, Trimetazidine; MCPA, 2-Methylene cyclopropane acetic acid; RNA-seq, RNA sequencing; MDS, multiple dimensional scaling; BrdU, 5-bromodeoxyuridine; PEMA, Preimplantation Embryos Metabolic Analysis; CUT&Tag, Cleavage Under Targets and Tagmentation.
